# Microbe-Specific C3b Deposition in the Horseshoe Crab Complement System in a C2/Factor B-Dependent or -Independent Manner

**DOI:** 10.1371/journal.pone.0036783

**Published:** 2012-05-07

**Authors:** Keisuke Tagawa, Toyoki Yoshihara, Toshio Shibata, Kazuki Kitazaki, Yuichi Endo, Teizo Fujita, Takumi Koshiba, Shun-ichiro Kawabata

**Affiliations:** 1 Graduate School of Systems Life Sciences, Kyushu University, Fukuoka, Japan; 2 Department of Biology, Faculty of Sciences, Kyushu University, Fukuoka, Japan; 3 Department of Immunology, Fukushima Medical University School of Medicine, Fukushima, Japan; Louisiana State University, United States of America

## Abstract

Complement C3 plays an essential role in the opsonization of pathogens in the mammalian complement system, whereas the molecular mechanism underlying C3 activation in invertebrates remains unknown. To understand the molecular mechanism of C3b deposition on microbes, we characterized two types of C2/factor B homologs (designated TtC2/Bf-1 and TtC2/Bf-2) identified from the horseshoe crab *Tachypleus tridentatus*. Although the domain architectures of TtC2/Bf-1 and TtC2/Bf-2 were identical to those of mammalian homologs, they contained five-repeated and seven-repeated complement control protein domains at their N-terminal regions, respectively. TtC2/Bf-1 and TtC2/Bf-2 were synthesized and glycosylated in hemocytes and secreted to hemolymph plasma, which existed in a complex with C3 (TtC3), and their activation by microbes was absolutely Mg^2+^-dependent. Flow cytometric analysis revealed that TtC3b deposition was Mg^2+^-dependent on Gram-positive bacteria or fungi, but not on Gram-negative bacteria. Moreover, this analysis demonstrated that Ca^2+^-dependent lectins (C-reactive protein-1 and tachylectin-5A) were required for TtC3b deposition on Gram-positive bacteria, and that a Ca^2+^-independent lectin (*Tachypleus* plasma lectin-1) was definitely indispensable for TtC3b deposition on fungi. In contrast, a horseshoe crab lipopolysaccharide-sensitive protease factor C was necessary and sufficient to deposit TtC3b on Gram-negative bacteria. We conclude that plasma lectins and factor C play key roles in microbe-specific TtC3b deposition in a C2/factor B-dependent or -independent manner.

## Introduction

Metazoans are endowed with a variety of host-defense mechanisms against invading pathogens. Innate immunity systems are essential for non-self recognition and for the clearance of pathogens from hosts. Pattern-recognition proteins, such as mammalian Toll-like receptors and *Drosophila* peptidoglycan-recognition proteins, recognize pathogen-associated molecular patterns (PAMPs), and activate signaling cascades that induce inflammation, phagocytosis, and subsequent adaptive immunity [1], [2]. The mammalian complement system is an important immune surveillance system involving more than 30 factors in plasma or on cytoplasmic membrane; complement C3 is activated via three convergent pathways (classical, alternative, and lectin) [3]. The activation of C3 can lead to a variety of host-defense systems, including the promotion of phagocytosis and the formation of a membrane-attack complex. The classical and lectin pathways are triggered by specific antibodies and pattern-recognition proteins such as mannose-binding lectin and ficolins, respectively. On the other hand, the alternative pathway is triggered by a small fraction of C3, in which the thioester bond is hydrolyzed to C3(H_2_O), exposing a binding site for complement factor B, a serine protease zymogen [4]. Factor B interacts with C3(H_2_O) in a Mg^2+^-dependent manner, and C3(H_2_O)B complex is proteolytically converted to C3(H_2_O)Bb (an initial C3 convertase) by complement factor D. The initial C3 convertase effectively cleaves C3 molecules into two fragments, C3a and C3b. The resulting C3a attracts phagocytes to an inflammation locus, and C3b binds to the surfaces of target cells, leading to C3bBb (a membrane-bound C3 convertase), which amplifies C3b deposition on the surfaces of target cells.

Zhu *et al.* identified homologs of mammalian C3 and C2/factor B from the Southeast Asian horseshoe crab *Carcinoscorpius rotundicauda*, designated CrC3 and CrC2/Bf, respectively, and reported that CrC3 and plasma lectins including Carcinolectins-5a and -5b (CL5a and CL5b) bind a wide range of microbes, forming the frontline innate immune system [5]. We previously demonstrated that C3 (TtC3), identified in the Japanese horseshoe crab *Tachypleus tridentatus*, is proteolytically activated by a lipopolysaccharide (LPS)-sensitive serine protease factor C in the presence of LPS, leading to C3b deposition on Gram-negative bacteria [6]. These results indicate that factor C acts as a TtC3 convertase on the surface of Gram-negative bacteria but not on that of Gram-positive bacteria or fungi. On the other hand, Ng *et al.* found, by using yeast two-hybrid and pull-down methods, that two serine proteases in *C. rotundicauda*, factor C and CrC2/Bf, can bind to three types of plasma lectins, including galactose-binding protein (CrGBP), Carcinolectin-5c (CL5c), and C-reactive protein-1 (CrCRP-1) [7]. In this study, we identified two structurally different types of C2/factor B in *T. tridentatus* (TtC2/Bf-1 and TtC2/Bf-2), and characterized whether the two complement factors participate in C3b deposition on microbes, including Gram-positive and -negative bacteria and fungi, in the presence or absence of plasma lectins.

## Results

### cDNA cloning of C2/factor B homologs from *T. tridentatus*

**Figure 1:**
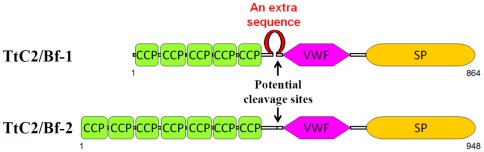
Schematic domain structures of TtC2/Bf-1 and TtC2/Bf-2. CCP, complement control protein domain; VWF, von Willebrand factor domain; SP, serine protease domain.

We identified a homolog of C2/factor B from *T. tridentatus* with 95.6% sequence identity to CrC2/Bf, designated TtC2/Bf-1 (Figure S1A, database accession no. AB 353280). In addition, we identified another type of C2/factor B, designated TtC2/Bf-2, by a specific set of polymerase chain reaction (PCR) primers for the amplification of the region around the active site His-57 in serine proteases (Figure S1B, database accession no. AB 353281). TtC2/Bf-1 and TtC2/Bf-2 consisted of 864 and 948 amino acid residues, respectively, which were composed of tandem-repeated complement control protein (CCP) domains, a von Willebrand factor (VWF) domain, and a trypsin-type serine protease (SP) domain (Figures 1 and S2). The domain architecture was the same as that of known C2/factor B homologs in mammals. Like CrC2/Bf [6] and sea urchin C2/factor B [8], TtC2/Bf-1 contained five-repeated CCPs (CCP1-CCP5), whereas TtC2/Bf-2 contained seven-repeated CCPs (CCP1-CCP7). The sequence identities between the corresponding domains of the two factors clearly showed that each domain of CCP1-CCP5 of TtC2/Bf-1 had the highest sequence identity to that of CCP3-CCP7 of TtC2/Bf-2 (Table 1). Interestingly, TtC2/Bf-1 contained an extra sequence of approximately 40 amino acids between CCP5 and VWF domains (Figure 1).

**Table 1:** Sequence identities among CCP, VWF and SP domains of TtC2/Bf-1 and TtC2/Bf-2.

Sequence identity (%)		TtC2/Bf-2								
		CCP1	CCP2	CCP3	CCP4	CCP5	CCP6	CCP7	VWF	SP
	CCP1	27.3	32.8	61.8	27.1	29.5	36.4	29.6		
	CCP2	31.8	25.8	28.3	76.3	27.9	35.6	30.5		
	CCP3	22.2	32.8	34.4	29.0	66.7	30.2	24.6		
TtC2/Bf-1	CCP4	21.0	27.9	32.1	28.8	28.3	81.8	49.1		
	CCP5	17.5	26.2	26.8	28.3	27.9	39.3	70.9		
	VWF								78.5	
	SP									66.7

CCP, complement control protein domain; VWF, von Willebrand factor domain; SP, serine protease domain.

### Tissue-specific localization of TtC2/Bf-1 and TtC2/Bf-2

**Figure 2:**
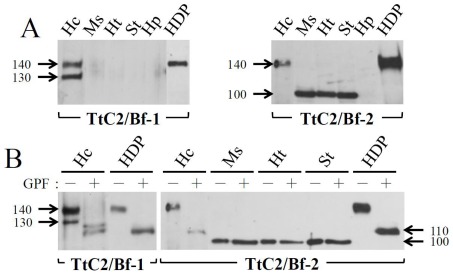
Tissue-specific localizations of TtC2/Bf-1 and TtC2/Bf-2 and the presence of their *N*-glycosylation forms. (A) Tissue extracts (10 µg each, quantified by micro BCA kit, Pierce) were subjected to Western blotting using the anti-TtC2/Bf-1-CCP antibody for TtC2/Bf-1 or the anti-TtC2/Bf-2-SP antibody for TtC2/Bf-2. Hc, hemocytes; Ms, muscle; Ht, heart; St, stomach; Hp, hepatopancreas; HDP, hemocyanin-depleted plasma. (B) Tissue extracts (2.5 µg each) were incubated with glycopeptidase F (GPF) at 37°C for 17 hours and subjected to Western blotting, as described in Figure 2A. Each experiment was performed at least three times. Representative blots are shown.

The tissue localization of TtC2/Bf-1 and TtC2/Bf-2 was evaluated by Western blotting using specific polyclonal antibodies: an anti-TtC2/Bf-1-CCP antibody for TtC2/Bf-1 and an anti-TtC2/Bf-2-SP antibody for TtC2/Bf-2 (Figure S3). For TtC2/Bf-1, a 140-kDa antigen was present in hemolymph plasma and hemocytes, and a lower molecular mass antigen of 130 kDa was also detected in hemocytes (Figure 2A). For TtC2/Bf-2, a 140-kDa antigen was detected in hemocytes and hemolymph plasma, and a lower molecular mass antigen of 100 kDa was detected in muscle, heart, and stomach but not in hemocytes. There were six and ten *N*-glycosylation potential sites in the amino acid sequences of TtC2/Bf-1 and TtC2/Bf-2, respectively (Figure S1), and treatment of the tissue extracts with glycopeptidase F converted the 140-kDa antigen in hemocytes and plasma to a 110-kDa antigen (Figure 2B). This indicated that the *N*-linked glycosylation was tissue-specific. These results indicate that the major expression sites of TtC2/Bf-1 and TtC2/Bf-2 are hemocytes, and that the glycosylated forms of TtC2/Bf-1 and TtC2/Bf-2 with 140 kDa are secreted from hemocytes to plasma. The mRNAs for TtC2/Bf-1 and TtC2/Bf-2 were detectable in hepatopancreas by reverse–transcription PCR (RT-PCR) (Figure S4), whereas these proteins were not detectable in this tissue by Western blotting (Figure 2A). The concentrations of TtC2/Bf-1 and TtC2/Bf-2 in hemolymph plasma were determined to be 0.24±0.04 mg/ml and 0.18±0.03 mg/ml, respectively, by enzyme-linked immunosorbent assay (ELISA).

### Activation of TtC2/Bf-1 and TtC2/Bf-2

**Figure 3:**
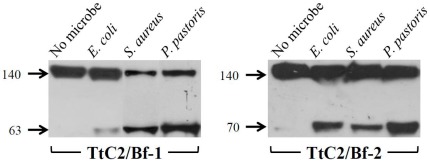
Microbe-induced activation of TtC2/Bf-1 and TtC2/Bf-2. Microbes were incubated with HDP at 37°C for 30 min. The microbes were removed by centrifugation, and 20 µl of the supernatants were subjected to Western blotting as described in Figure 2A. Lane 1, HDP; lane 2, HDP+*E. coli*; lane 3, HDP+*S. aureus*; lane 4, HDP+*P. pastoris*. Each experiment was performed at least three times. Representative blots are shown.

To evaluate whether or not microbes trigger the activation of TtC2/Bf-1 and TtC2/Bf-2, hemocyanin-depleted hemolymph plasma (HDP) was incubated with Gram-negative bacteria (*Escherichia coli*), Gram-positive bacteria (*Staphylococcus aureus*), and fungi (*Pichia pastoris*). The conversion of TtC2/Bf-1 and TtC2/Bf-2 to their active forms was analyzed by Western blotting. HDP was used for these experiments because a large amount of hemocyanin subunits with 70 kDa interrupted the detection of the activation fragments derived from TtC2/Bf-1 and TtC2/Bf-2. TtC2/Bf-1 and TtC2/Bf-2 were converted to the active forms by mixing with microbes (Figure 3). The anti-TtC2/Bf-1-CCP antibody recognized the N-terminal part of TtC2/Bf-1 with 63 kDa (Figure 3, left panel), and the C-terminal proteolytic fragment of TtC2/Bf-1 with 74 kDa was detected with another anti-TtC2/Bf-1-SP antibody (Figure S5). On the other hand, the anti-TtC2/Bf-2-SP antibody recognized the C-terminal proteolytic fragment of TtC2/Bf-2 with 70 kDa (Figure 3, right panel).

**Figure 4:**
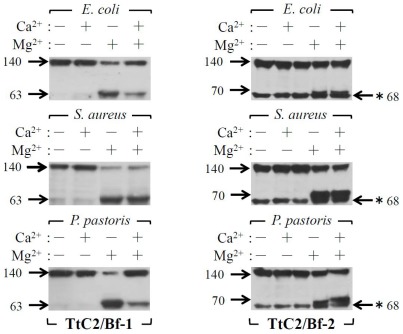
Mg^2+^-dependent activation of TtC2/Bf-1 and TtC2/Bf-2. Microbes were incubated with dialyzed HDP at 37°C for 30 min in the presence (CaCl_2_ = 10 mM, MgCl_2_ = 50 mM) or absence of divalent cations. Microbes were removed by centrifugation, and 20 µl of the supernatants were subjected to Western blotting, as described in Figure 2A. Each experiment was performed at least three times. Representative blots are shown. A cross-reacting protein with 68 kDa against the anti-TtC2/Bf-2-SP antibody by Western blotting is shown by (*).

**Figure 5:**
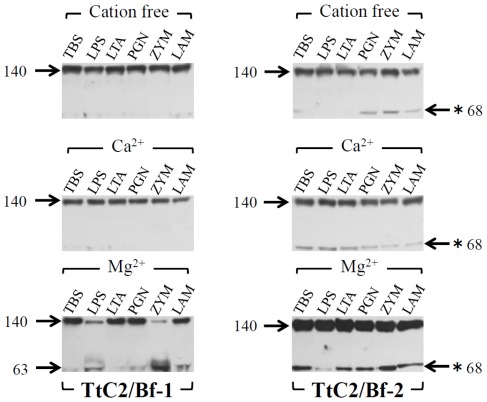
PAMPs-induced activation of TtC2/Bf-1 and TtC2/Bf-2. Pathogen-associated molecular patterns (PAMPs) were incubated with 20 µl of dialyzed HDP at 37°C for 30 min in the presence or absence of cations and then subjected to Western blotting, as described in Figure 2A. TBS, Tris-buffered saline; LPS, lipopolysaccharide; LTA, lipoteichoic acid; PGN, peptidoglycan; ZYM, zymosan; LAM, laminarin. Each experiment was performed at least three times. Representative blots are shown. A cross-reacting protein with 68 kDa against the anti-TtC2/Bf-2-SP antibody by Western blotting is shown by (*).

**Figure 6:**
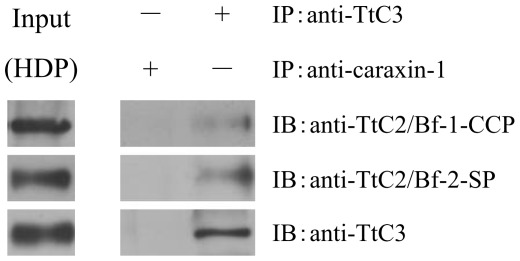
Interaction of TtC2/Bf-1 or TtC2/Bf-2 with TtC3. TtC3 in 20 µl of HDP was immunoprecipitated with anti-TtC3 antibody or control antibody (anti-caraxin-1 antibody) at room temperature for 30 min. TtC2/Bf-1 and TtC2/Bf-2 interactions with TtC3 were detected by Western blotting, as described in Figure 2A. Two µl of HDP was loaded as 10% input. Each experiment was performed at least three times. Representative blots are shown.

The proteolytic activation of factor B by factor D in the mammalian complement system requires Mg^2+^ ions that bind to factor B to induce a conformational change, leading to the complex formation with C3 [4]. We prepared cation-free HDP by dialysis. As expected, the activation of TtC2/Bf-1 and TtC2/Bf-2 by microbes was absolutely Mg^2+^-dependent (Figure 4). In these experiments, the anti-TtC2/Bf-2-SP antibody, but not the two types of anti-TtC2/Bf-1 antibodies, cross-reacted with an unknown protein with apparent molecular mass of 68 kDa, and the cross-reacting protein was not a derivative from TtC2/Bf-2, since the 68-kDa protein was observed in the absence of Mg^2+^ ions (Figure 4, right panels). The mammalian complement system is also activated by PAMPs, such as LPS of Gram-negative bacteria, and by zymosan of fungi [2], [3]. HDP in the absence or presence of cations was incubated with PAMPs including LPS, lipoteichoic acid, peptidoglycan, zymosan, and laminarin (Figure 5). Zymosan, laminarin, and LPS effectively activated TtC2/Bf-1 in the presence of Mg^2+^, but none of the PAMPs could activate TtC2/Bf-2. In order to know whether TtC2/Bf-1 or TtC2/Bf-2 is present as a complex with TtC3 in hemolymph plasma, HDP was mixed with the anti-TtC3 antibody and immunoprecipitated by protein G Sepharose. TtC2/Bf-1 and TtC2/Bf-2 were detected in the resulting immunoprecipitates by Western blotting, revealing that TtC2/Bf-1 and TtC2/Bf-2 are present in the complex form with TtC3 (Figure 6).

### Mg^2+^-dependent TtC3b deposition on Gram-positive bacteria and fungi

**Figure 7:**
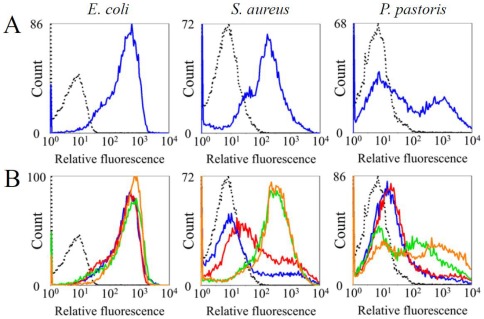
Effects of Mg^2+^ and Ca^2+^ on the deposition of TtC3b on microbes. (A) Microbes were incubated with HDP. The deposition of TtC3b on the microbial surfaces was analyzed by flow cytometry using the Alexa 488-conjugated anti-TtC3 antibody (blue line). The dotted line indicates autonomous fluorescence (AF) of microbes. (B) Dialyzed HDP was incubated with various microbes, and the deposition of TtC3b on the microbial surfaces was analyzed in the presence or absence of cations, as described in Figure 7A. Blue line, dialyzed HDP; red line, dialyzed HDP+10 mM Ca^2+^; green line, dialyzed HDP+50 mM Mg^2+^; orange line, dialyzed HDP+10 mM Ca^2+^ and 50 mM Mg^2+^. Mean fluorescence intensity of *E. coli*: AF, 5.9; HDP, 250; dialyzed HDP, 266; dialyzed HDP+Ca^2+^, 236; dialyzed HDP+Mg^2+^, 286; dialyzed HDP+Ca^2+^ and Mg^2+^, 376. MFI of *S. aureus*: AF, 6.8; HDP, 116; dialyzed HDP, 21; dialyzed HDP+Ca^2+^, 62; dialyzed HDP+Mg^2+^, 220; dialyzed HDP+Ca^2+^ and Mg^2+^, 231. MFI of *P. pastoris*: AF, 6.1; HDP, 57; dialyzed HDP, 20; dialyzed HDP+Ca^2+^, 28; dialyzed HDP+Mg^2+^, 60; dialyzed HDP+Ca^2+^ and Mg^2+^, 139. In (A) and (B), one representative experiment out of three repeats is shown.

Opsonization by C3b is one of the most important responses in the mammalian complement system [2], [3]. To evaluate the opsonization ability of HDP, *E. coli*, *S. aureus*, and *P. pastoris* were mixed with HDP, and TtC3b deposition on microbes was quantified by flow cytometric analysis. The three types of microbes were opsonized with TtC3b (Figure 7A). TtC3b deposition was relatively homogeneous on the surface of *E. coli* and *S. aureus*, whereas a bimodal distribution of TtC3b was observed on the surface of *P. pastoris*. At present, the reason for this discrepancy is unclear.

TtC3b deposition on *S. aureus* and *P. pastoris* was strongly inhibited under cation-free conditions, whereas TtC3b deposition on *E. coli* was not influenced under the same conditions (Figure 7B, blue lines). The supplementation of Mg^2+^ effectively restored the ability for TtC3b deposition on *S. aureus* and *P. pastoris* (Figure 7B, green lines). On the other hand, the supplementation of Ca^2+^ partially restored TtC3b deposition on *S. aureus*, but had little effect on the TtC3b deposition on *P. pastoris* (Figure 7B, red lines). These observations demonstrate that the Mg^2+^-dependent activation of TtC2/Bf-1 or TtC2/Bf-2 is essentially required for TtC3b deposition on Gram-positive bacteria and fungi, and suggest that Ca^2+^-dependent lectins enhance TtC3b deposition on Gram-positive bacteria. In contrast, the activation of TtC2/Bf-1 or TtC2/Bf-2 and the participation of Ca^2+^-dependent proteins were not essential for TtC3b deposition on Gram-negative bacteria.

**Figure 8:**
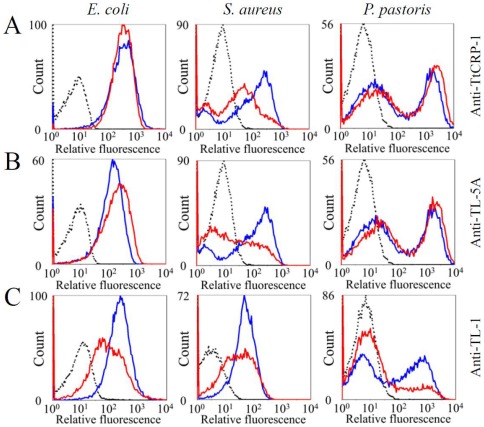
Effects of plasma lectins on the deposition of TtC3b on microbes. Microbes were incubated with HDP, and the deposition of TtC3b on the microbial surface was analyzed by flow cytometry, as described in Figure 7A (blue line). The dotted line indicates autonomous fluorescence. The red line indicates flow-cytometric data obtained by pretreatment of HDP with the anti-TtCRP-1 (*Tachypleus tridentatus* C-reactive protein-1) antibody (A), anti-TL-5A (Tachylectin-5A) antibody (B), or anti-TL-1 (Tachylectin-1) antibody (C) at 10 µg/ml for 1 hour at 4°C. Mean fluorescence intensity of *E. coli*: (A) AF, 7.2; HDP, 266; HDP+antibody, 276; (B) AF, 6.83; HDP, 110; HDP+antibody, 155; (C) AF, 8.96; HDP, 201; HDP+antibody, 79. MFI of *S. aureus*: (A) AF, 7.2; HDP, 66; HDP+antibody, 26; (B) AF, 7.2; HDP, 66; HDP+antibody, 18; (C) AF, 3.6; HDP, 42; HDP+antibody, 25. Mean fluorescence intensity of *P. pastoris*: (A) AF, 5.8; HDP, 96; HDP+antibody, 160; (B) AF, 5.8; HDP, 96; HDP+antibody, 150; (C) AF, 6.04; HDP, 50; HDP+antibody, 16. In (A), (B), and (C), one representative experiment out of three repeats is shown.

In *T. tridentatus* hemolymph plasma, there are several types of Ca^2+^-dependent lectins, such as C-reactive protein-1 (TtCRP-1, a homolog to CrCRP-1) [9] and tachylectin-5A (TL-5A, a homolog to CL5a) [10]. To evaluate whether or not the Ca^2+^-dependent lectins are involved in the enhancement of TtC3b deposition, microbes were incubated with HDP pretreated with antibodies against TtCRP-1 and TL-5A. These antibodies effectively inhibited TtC3b deposition on *S. aureus* but not on *E. coli* or *P. pastoris* (Figures 8A and 8B). On the other hand, a Ca^2+^-independent lectin has been identified in plasma, *Tachypleus* plasma lectin-1 with 26 kDa (TPL-1, an ortholog of CrGBP) [11]–[13]. A polyclonal antibody previously prepared against a hemocyte-derived tachylectin-1 (TL-1, an isolectin for TPL-1) [14] had cross-reactivity against TPL-1 and its isolectins with 25∼27-kDa in hemolymph plasma (Figure S6). HDP was treated with the anti-TL-1 antibody and was mixed with microbes. TtC3b deposition was inhibited almost completely on *P. pastoris* and was inhibited partially on *E. coli* and *S. aureus* (Figure 8C), suggesting that TPL-1 has an essential role in TtC3b deposition on fungi.

## Discussion

Here we characterized the two types of C2/factor B (TtC2/Bf-1 and TtB2/Bf-2) identified in the horseshoe crab *T. tridentatus*. TtC2/Bf-1 and TtC2/Bf-2 contained five-repeated and seven-repeated CCPs at their N-terminal regions, respectively. Based on the sequence homology between the two factors (Table 1), we suppose that a gene duplication of an ancestor C2/factor B produced two isoforms, following the linkage of two additional CCPs to the N-terminus of one of the paralogs to produce a gene for TtC2/Bf-2.

Both TtC2/Bf-1 and TtC2/Bf-2 were synthesized and highly glycosylated in hemocytes and were secreted to hemolymph plasma with an apparent *Mr.* = 140 kDa (Figures 2A and 2B). Interestingly, the lower molecular mass form of TtC2/Bf-2 with 100 kDa was detected in muscle, heart, and stomach by Western blotting (Figure 2A), and its physiological role in these tissues remains unknown. TtC2/Bf-1 and TtC2/Bf-2 were proteolytically cleaved in a Mg^2+^-dependent manner in the presence of microbes (Figures 3 and 4). Judging from the sequence around the cleavage site of factor B by factor D in the mammalian complement system, we estimate the potential cleavage sites of –Arg^390^-X- for TtC2/Bf-1 and of -Arg^472^-X- for TtC2/Bf-2 (Figure 1), although a factor D-like serine protease for their activation has not been identified in the horseshoe crab.

TtC2/Bf-1 or TtC2/Bf-2 was effectively activated in the presence of microbes including *E. coli*, *S. aureus*, and *P. pastoris*, whereas PAMPs such as lipoteichoic acid and peptidoglycan derived from Gram-positive bacteria had little or no effect on the activation of TtC2/Bf-1, and none of the PAMPs activated TtC2/Bf-2 (Figure 5). Native insoluble structures of PAMPs on the surfaces of microbes are necessary for the effective activation of TtC2/Bf-1 and TtC2/Bf-2, but their extracted soluble forms are not.

Immunoprecipitation and Western blotting showed that TtC2/Bf-1 or TtC2/Bf-2 interacts with TtC3 in hemolymph plasma (Figure 6). TtC3 is present in hemolymph plasma at concentrations >0.3 mg/ml [6], and the concentration of TtC2/Bf-1 or TtC2/Bf-2 was determined to be ∼0.2 mg/ml. It is therefore possible that a portion of TtC2/Bf-1 or TtC2/Bf-2 in hemolymph plasma may exist continually in a complex with TtC3 and that the resulting complex is a prerequisite for the immediate activation of the complement system on the surfaces of microbes.

Zhu e*t al.* observed LPS-induced serine protease activity in *C. rotundicauda* plasma in a Mg^2+^ and Ca^2+^ -dependent manner, and suggested that there must be an alternative or a lectin pathway in mammals [5]. Ng *et al.* also reported that factor C and CrC2/Bf can bind to plasma lectins, including CrGBP, CL5c, and CrCRP-1, by using yeast two-hybrid and pull-down methods [7]. Our flow cytometric analyses showed that TtC3b deposition on Gram-positive bacteria and fungi, but not on Gram-negative bacteria, was absolutely Mg^2+^-dependent (Figure 7B). This indicated that TtC2/Bf-1 or TtC2/Bf-2 is indispensable for TtC3b deposition on Gram-positive bacteria and fungi. Using specific polyclonal antibodies, we revealed that Ca^2+^-dependent plasma lectins, TtCRP-1 (an ortholog of CrCRP-1) and TL-5A (an ortholog of CL5c), were required for TtC3b deposition on Gram-positive bacteria in the horseshoe crab complement system (Figures 8A and 8B). TtCRP-1 is one of the most abundant plasma CRPs in the horseshoe crab [9], and TL-5A recognizes an acetyl group and has agglutinating activity against Gram-positive bacteria [10]. Together, these may work as pattern-recognition proteins for Gram-positive bacteria. TtCRP-1 and TL-5A are also homologs of mammalian CRP and ficolins, which can activate the complement classical and lectin pathways in mammals. Moreover, a Ca^2+^-independent plasma lectin, TPL-1 (an ortholog of CrGBP), was definitely indispensable for TtC3b deposition on fungi (Figure 8C). In response to LPS stimulation, hemocytes secrete TL-1 (an isolectin of TPL-1), one of the most abundant hemocyte-derived lectins [14]. Therefore, TL-1 may be involved in TtC3b deposition on fungi.

**Figure 9:**
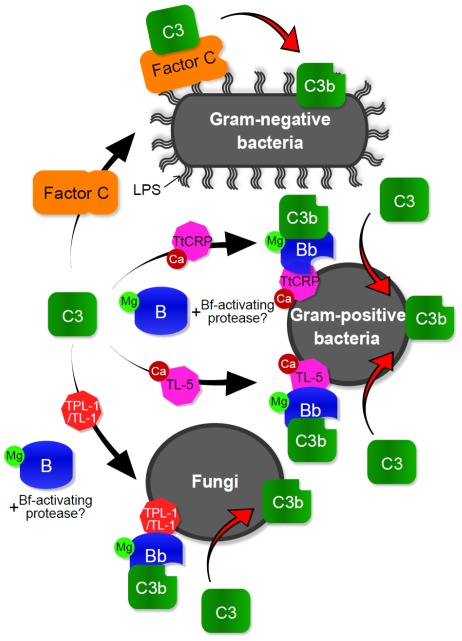
Proposed mechanism for deposition of TtC3b on the surfaces of microbes. On the surface of Gram-negative bacteria, the LPS-sensitive protease factor C is activated through the interaction with LPS. Activated factor C then acts as a C3 convertase, and the resulting TtC3b is deposited. On the Gram-positive bacteria, Ca^2+^-dependent lectins such as TtCRP-1 (*Tachypleus tridentatus* C-reactive protein-1) and TL-5A (Tachylectin-5A) recruit the complex (C3-B), TtC3-TtC2/Bf-1, or TtC3-TtC2/Bf-2. The second C3 convertase (C3b-Bb), TtC3b-TtC2/Bf-1b or TtC3b-TtC2/Bf-2b complex, may be formed by an unidentified protease on the surfaces of Gram-positive bacteria and fungi. TL-1 (Tachylectin-1) and possibly TPL-1 (*Tachypleus* plasma lectin-1) also promote TtC3b deposition on the three types of microbes, especially on fungi.

Figure 9 shows a schematic model of TtC3b deposition on the surfaces of three types of microbes. The Ca^2+^-dependent lectins (TtCRP-1 and TL-5A) and the Ca^2+^-independent lectin (TLP-1/TL-1) seem to recruit the complex between TtC3 and TtC2/Bf-1 or TtC2/Bf-2 (C3(H_2_O)B) on Gram-positive bacteria and fungi, respectively, to convert the complex to a TtC3 convertase (C3bBb). These lectins seem to work as carries for the C3bBb-like convertase to the surface of microbes with quite specific binding activities. However, these lectins have broad binding specificities against microbial cell wall substances and bind not only to Gram-positive bacteria but also to Gram-negative bacteria [7], [9], [10], [13], [14]. In addition, TPL-1 and TL-1 bind to saccharides, such as *N*-acetyl-glucosamine, galactose, and mannan [13], [14], and possibly bind to β-1,3-D-glucan, a major cell wall component of fungi. Therefore, the binding specificities of these lectins are not sufficient to explain the microbe specific TtC3b deposition. Unknown proteins may be additionally required to determine the specific target of these lectins.

Although TtC2/Bf-1 or TtC2/Bf-2 was activated in the presence of LPS (Figure 5), the C3bBb-like convertase could not be effectively recruited by the horseshoe crab lectins to the surface on Gram-negative bacteria. Our previous results demonstrated that factor C exists in a complex with TtC3 (*K*_d_ = 4.9×10^−8^ M), a prerequisite for the immediate activation of TtC3 on Gram-negative bacteria [6]. This suggested that factor C recruits TtC3 to the surface of Gram-negative bacteria for TtC3b deposition. We conclude that the horseshoe crab lectins and factor C play key roles in microbe-specific TtC3b deposition in a C2/factor B-dependent or -independent manner.

## Materials and Methods

### Materials

*T. tridentatus* horseshoe crabs were collected in Hakata Bay, Japan, and maintained in aerated tanks with running seawater at 23°C. Benzamidine, peptidoglycan (*S. aureus*), laminarin (*Laminaria digitata*), and lipoteichoic acid (*S. aureus*) were from Sigma-Aldrich. LPS (*Salmonella minnesota* R595) was from List Biological Laboratories. Polyclonal antibodies against TtC3, tachylectin-5A, TtCRP-1, and caraxin-1 were prepared previously [6], [9], [10], [15].

### cDNA cloning of TtC2/Bf-1 and TtC2/Bf-2

The degenerate nucleotide sequences of the primers used for RT-PCR with cDNA of the hepatopancreas or muscle as a template were based on the amino acid sequences of CrC2/Bf [5]. The conditions used for RT-PCR and 5′- or 3′-RACE (rapid amplification of cDNA ends) analysis were as described previously [16]. TtC2/Bf-2 was obtained by amplification using the cDNA of hepatopancreas as a template and degenerated primers that were designed based on the conserved sequences of the serine protease superfamily: CxPxCG (F, 5′-TGYIWDCCWRHITGYGG-3′), TAAHxx (R2, 5′-ACVIMRTGDGCIGCYGT-3′), and MxCAGx (R1, 5′-TMBCCRGCRCARAWCAT-3′). The PCR was nested using primers F and R1 for the first amplification and F and R2 for the second amplification. A 0.2 Kb product was cloned into a pGEM-T easy vector (Clontech, Palo Alto, CA) and sequenced. To complete the cDNA sequence, 5′-and 3′-RACE were performed using a Marathon kit (Clontech). The resulting cDNA sequences are shown in Figures S1A and S1B.

### Preparation of specific polyclonal antibodies against TtC2/Bf-1 and TtC2/Bf-2

Each domain of TtC2/Bf-1 and TtC2/Bf-2, including CCP domains (TtC2/Bf-1-CCP, residue nos. 1–312) and SP domain (TtC2/Bf-1-SP, nos. 737–864 and TtC2/Bf-2-SP, nos. 687–939) was expressed in *E. coli* and used for the preparation of polyclonal antibodies. To construct expression vectors, cDNA fragments encoding these domains were amplified by PCR. An amplified cDNA fragment encoding TtC2/Bf-1-SP was cloned via *Nco*I and *EcoR*I sites into expression vector pET-28b (Novagen). TtC2/Bf-1-CCP was cloned via *Nde*I and *Xho*I sites into expression vector pET-22b (Novagen). TtC2/Bf-2-SP was cloned via *Nco*I and *BamH*I sites into expression vector pET-15b (Novagen). All constructs were verified by sequencing. These constructs were expressed in the *Escherichia coli* strain BL21 (DE3)/pLysS. Bacteria were cultured in Luria-Bertani medium, induced by the addition of isopropyl-β-D-thiogalactoside to a final concentration of 1 mM, and incubated at 37°C for 3 h. Then, bacteria were sonicated in 10 ml of sonication buffer (20 mM Tris-HCl (pH 8.0) containing 1 mM EDTA). After the sonication, the precipitates were recovered by centrifugation, washed three times with 4 ml of sonication buffer, and dissolved in 4 ml of 20 mM Tris-HCl (pH 8.0) containing 8 M urea. This solution was then subjected to SDS-PAGE under reducing conditions and then stained using negative staining. The protein bands corresponding to the recombinant proteins were excised from the gel and recovered by electroelution for the immunization of rabbits (KBT Oriental, Saga, Japan). Antibodies were purified from each anti-serum by using protein A-Sepharose and antigen-immobilized gels prepared with the corresponding antigens and affi-Gel10 (Bio-Rad). The resulting antibodies, including anti-TtC2/Bf-1-CCP, anti-TtC2/Bf-1-SP, and anti-TtC2/Bf-2-SP, specifically recognized the antigens.

### Preparation of HDP

Hemolymph plasma was collected from a single animal into a sterilized plastic tube and centrifuged at 50× g for 10 min to remove hemocytes. The resulting plasma was further centrifuged at 100,000× g for 4 h to remove hemocyanin and dialyzed against 20 mM Tris-HCl (pH 7.5) containing 0.15 M NaCl and 1 mM EDTA. For complement activation, HDP was supplemented with 10 mM CaCl_2_ and 50 mM MgCl_2_, equivalent to the concentrations in horseshoe crab hemolymph plasma [17].

### Extraction of RNA from various tissues and RT-PCR

mRNA was extracted from the hepatopancreas of *T. tridentatus* using RNAiso (Takara) and Oligotex™-dT30 mRNA Purification Kit (Takara). mRNAs from the other tissues were extracted using QuickPrep mRNA purification kit (GE Healthcare). First-strand cDNA synthesis was performed using SuperScript III RNase H^−^ reverse transcriptase (Invitrogen). The resulting cDNA and each primer were subjected to PCR (35 cycles) with denaturation at 95°C for 5 s, annealing at 60°C for 10 s, and extension at 72°C for 30 s. PCR products were analyzed on a 2% agarose gel and visualized following ethidium bromide staining.

### Immunoprecipitation assay

IgGs were added to 20 µl of HDP and incubated at room temperature for 30 min. Immunoprecipitation was performed with Protein G Sepharose™ 4 Fast Flow (GE Healthcare). Beads were washed three times each with 20 mM Tris-HCl (pH 7.5) containing 0.15 M NaCl, and IgGs were eluted by incubation with the sampling buffer for SDS-PAGE. The resulting samples were subjected to SDS-PAGE under the reducing condition and to Western blotting.

### Flow cytometric analysis

*E. coli* K12, *S. aureus* p209, and *P. pastoris* X33 were incubated with hemolymph plasma at 37°C for 30 min. After being washed with 10 mM sodium phosphate (pH 7.0) containing 0.05% SDS and then with the same buffer without SDS, the microorganisms were incubated with the Alexa 488-conjugated anti-TtC3 antibody at 37°C for 30 min. After washing with buffer, the labeled microorganisms were analyzed by a flow cytometer (Beckman Coulter, EPICS XL System).

### SDS-PAGE and Western blotting

SDS-PAGE was performed in 6, 8, or 10% slab gel, according to Laemmli [18]. Western blotting was performed by standard procedures. Precision Plus Protein Standards (Bio-Rad) were used to determine apparent molecular masses. Samples were subjected to SDS-PAGE and transferred to a PVDF membrane. After blocking with 5% skim milk, the membrane was incubated with anti-TtC2/Bf-1-CCP antibody or anti-TtC2/Bf-2-SP antibody and then with the secondary antibody (horseradish peroxidase-conjugated goat anti-rabbit IgG, Bio-Rad), followed by development with Chemi-Lumi One (Nacalai Tesque).

### ELISA

Microtiter plates were coated with the anti-TtC2/Bf-1-CCP antibody or anti-TtC2/Bf-2-SP antibody (10 µg/ml, 50 µl) by incubation at 37°C for 1 h. After washing with 20 mM Tris-HCl, pH 7.5, containing 0.15 M NaCl, the plates were blocked with 2.5% casein in the same buffer, and two-fold serial dilutions (50 µl each) of HDP or purified TtC2/Bf-1 antigen or TtC2/Bf-2 antigen to make a standard curve were added, incubated at 37°C for 1 h, and then washed. The biotinylated anti-TtC2/Bf-1-CCP antibody or anti-TtC2/Bf-2-SP antibody (1 µg/ml, 50 µl) was added, incubated at 37°C for 1 h, and washed. The 500-fold diluted avidin-HRP (Bio-Rad) was added and incubated at 37°C for 1 h. The enzyme activity of horseradish peroxidase was detected with o-phenylenediamine at 492 nm by using a microplate reader, model 3550 (Bio-Rad).

## Supporting Information

Figure S1 **The cDNA (upper) and deduced amino acid sequences (lower) of TtC2/Bf-1 (A) and TtC2/Bf-2 (B).** Nucleotides are numbered on the right. The amino acid sequence is numbered beginning at the N-terminus of the mature TtC2/Bf-1 or TtC2/Bf-2. The domain arrangements are shown in boxes. Tryp_SP, trypsin-like serine protease domain. Potential *N*-glycosylation sites are marked by blue diamonds.
(TIF)

Figure S2 **The sequence comparison between TtC2/Bf-1 and TtC2/Bf-2.**
(TIF)

Figure S3 **Anti-TtC2/Bf-1-CCP and anti-TtC2/Bf-2-SP antibodies specifically detect plasma TtC2/Bf-1 and TtC2/Bf-2, respectively.** Anti-TtC2/Bf-1-CCP antibody was not neutralized with TtC2/Bf-2 antigen (lane 3) but was with TtC2/Bf-1 antigen (lane 2). Anti-TtC2/Bf-2-SP antibody was not neutralized with TtC2/Bf-1 antigen (lane 5) but was with TtC2/Bf-2 antigen (lane 6). Lane 1, anti-TtC2/Bf-1-CCP antibody; lane 2, anti-TtC2/Bf-1-CCP antibody+TtC2/Bf-1 antigen; lane 3, anti-TtC2/Bf-1-CCP antibody+TtC2/Bf-2 antigen; lane 4, anti-TtC2/Bf-2-SP antibody; lane 5, anti-TtC2/Bf-2-SP antibody+TtC2/Bf-1 antigen; lane 6, anti-TtC2/Bf-2-SP antibody+TtC2/Bf-2 antigen. One representative experiment out of three repeats is shown.
(TIF)

Figure S4 **Expression patterns of TtC2/Bf-1 and TtC2/Bf-2.** Relative mRNA expression patterns of TtC2/Bf-1 and TtC2/Bf-2 were investigated by RT-PCR. Hc, hemocytes; Ms, muscle; Ht, heart; St, stomach; Hp, hepatopancreas. The relative mRNA expression pattern of big defensin was used as a positive control. One representative experiment out of three repeats is shown.
(TIF)

Figure S5 **Detection of the two activation fragments of TtC2/Bf-1 by specific polyclonal antibodies.** TtC2/Bf-1 was incubated with *P. pastoris* and subjected to Western blotting using the anti-TtC2/Bf-1-CCP antibody for the N-terminal fragment or the anti-TtC2/Bf-1-SP antibody for the C-terminal fragment. Lane 1, plasma; lane 2, plasma+*P. pastoris*. One representative experiment out of three repeats is shown.
(TIF)

Figure S6 **Western blotting of hemolymph plasma against the anti-TL-1 antibody.** One representative experiment out of three repeats is shown.
(TIF)
